# Efficacy and safety of curcuminoids alone in alleviating pain and dysfunction for knee osteoarthritis: a systematic review and meta-analysis of randomized controlled trials

**DOI:** 10.1186/s12906-022-03740-9

**Published:** 2022-10-19

**Authors:** Jie Feng, Zhao Li, Linling Tian, Panyun Mu, Yimei Hu, Feng Xiong, Xu Ma

**Affiliations:** 1 Department of Clinical Medicine, Chengdu University of Traditional Chinese Medicine, Chengdu, Sichuan Province China; 2 Department of Orthopedics, Affiliated Hospital of Chengdu University of Traditional Chinese Medicine, Chengdu, Sichuan Province China

**Keywords:** Curcuminoids, Knee osteoarthritis, Clinical effectiveness, Minimum clinically important difference, Systematic review, Meta-analysis

## Abstract

**Background:**

Curcuminoids (CURs) are the principal ingredients of *Curcuma longa L. [Zingiberaceae]* (CL)—an herbal plant used in east Asia to alleviate pain and inflammation. Thus far, the therapeutic effects of CURs for knee osteoarthritis (OA) uncovered by multiple reviews remained uncertain due to broadly involving trials with different agents-combined or CURs-free interventions. Therefore, we formed stringent selection criteria and assessment methods to summarize current evidence on the efficacy and safety of CURs alone in the treatment of knee OA.

**Methods:**

A series of databases were searched for randomized controlled trials (RCTs) evaluating the efficacy and safety of CURs for knee OA. Clinical outcomes were evaluated using meta-analysis and the minimum clinically important difference (MCID) for both statistical and clinical significance.

**Results:**

Fifteen studies with 1670 patients were included. CURs were significantly more effective than placebo in the improvements of VAS for pain ( WMD: − 1.77, 95% CI: − 2.44 to − 1.09), WOMAC total score ( WMD: − 7.06, 95% CI: − 12.27 to − 1.84), WOMAC pain score ( WMD: − 1.42, 95% CI: − 2.41 to − 0.43), WOMAC function score ( WMD: − 5.04, 95% CI: − 7.65 to − 2.43), and WOMAC stiffness score ( WMD: − 0.54, 95% CI: − 1.03 to − 0.05). Meanwhile, CURs were not inferior to NSAIDs in the improvements of pain- and function-related outcomes. Additionally, CURs did not significantly increase the incidence of adverse events (AEs) compared with placebo ( RR: 1.03, 95% CI: 0.69 to 1.53, *P* = 0.899, I^2^ = 23.7%) and NSAIDs (RR: 0.71 0.65, 95% CI: 0.57 0.41 to 0.90 1.03).

**Conclusions:**

CURs alone can be expected to achieve considerable analgesic and functional promotion effects for patients with symptomatic knee OA in short term, without inducing an increase of adverse events. However, considering the low quality and substantial heterogeneity of present studies, a cautious and conservative recommendation for broader clinical use of CURs should still be made. Further high-quality studies are necessary to investigate the impact of different dosages, optimization techniques and administration approaches on long-term safety and efficacy of CURs, so as to strengthen clinical decision making for patients with symptomatic knee OA.

**Supplementary Information:**

The online version contains supplementary material available at 10.1186/s12906-022-03740-9.

## Introduction

Knee osteoarthritis (OA) is one of the most general irreversible articulus diseases globally, and presents with features of incremental cartilage defect and articular space narrowing. Approximately 11.8% to 12.7% of the global population are affected by knee OA, according to World Health Organization [1]. The condition is similar in China, where the number of OA patients nearly increased 2.35-fold over the past three decades, and approximately 61.2 million individuals suffered from symptomatic OA in 2017, with a percentage of mild, moderate, and severe OA of 47%, 35.9%, and 17.1% respectively [2]. Despite the high prevalence of knee OA, effective and permanent interventions to halt or reverse the degenerative progression have not yet been developed [3]. Intra-articular chronic inflammation accompanied with joint pain and dysfunction is the main pathological features of knee OA, which necessitate long-term management. Widely applied pharmacotherapies aimed at anti-inflammation and pain reduction are limited to acetaminophen and non-steroidal anti-inflammatory drugs (NSAIDs) [4]. While conventional medications have only a marginal effect on pain, with no significant impact on joint function. Adverse events (AEs) that may occur in digestive and cardiovascular systems also restrict the feasibility of long-term administration of NSAIDS [5–7]. Hence, the exploration of alternative options with good safety and efficacy profiles for knee OA has been delved into traditional herbal medicine [8–10]. Notably, curcumin, extracted from the rhizome of Curcuma longa L. [Zingiberaceae] (CL), is a botanical extract with promising clinical values [11].

Analogues comprising curcumin, bisdemethoxycurcumin, demethoxycurcumin and cyclocurcumin are collectively referred to as curcuminoids (CURs) [12], which constitute the principal ingredients of CL—an herbal plant used in east Asia to alleviate pain and inflammation. CURs are natural polyphenols which have been shown to exert anti-inflammatory and anti-oxidant effects in vivo and vitro studies by downregulating inflammation-related nuclear factor kappa-B (NF-κB) signaling pathway, scavenging free radicals, and inhibiting the activity of enzymes, such as cyclooxygenase-2 (COX-2), 5-lipoxygenase (5-LOX), and nitric oxide synthase (NOS), which exacerbate the oxidative stress in OA condition [13–15]. Normal NSAIDS are of critical safety concerns due to simultaneously inhibition of COX-1 and COX-2 enzymes in arthritis, while CURs can reduce the synthesis of COX-2 tendentiously [16], which may result in better safety profiles. Furthermore, CURs exhibit chondroprotective properties by stimulating extracellular matrix synthesis, down-regulating the synthesis of matrix metalloproteinases (MMPs) [17]. And CURs were shown to postpone joint contracture progress via inhibiting the proliferation of myofibroblasts from the joint capsule [18]. Considering that the pathophysiology of knee OA is characterized by inflammation and degeneration with prominent symptoms of pain and dysfunction, alleviating local inflammation and oxidative stress, stimulating cartilage regeneration and delaying joint contracture may be conducive to the condition, and CURs have emerged as an attractive treatment option for knee OA.

Several animal studies have assessed the efficacy of CURs administered via nano-scale drug carriers for knee OA, demonstrating that CURs have potent anti-inflammatory and anti-arthritic activity, both with and without biological materials [19–23]. Although pre-clinical studies have revealed promising results, the clinical efficacy, safety, dosage, and treatment duration of CURs for knee OA remain equivocal. Thus far, the therapeutic effects of CURs for knee osteoarthritis (OA) uncovered by multiple reviews remained uncertain due to broadly involving trials with different agents-combined or CURs-free interventions [8, 24–26], and evidence to reveal the clinical significance of CURs alone for knee OA is insufficient. Consequently, we aimed to summarize the evidence to date on the clinical effectiveness of CURs alone in alleviating pain and dysfunction for knee OA by a systematic review and meta-analysis. We postulated that CURs have superior efficacy in pain relief and functional promotion compared to control measures.

## Methods

The research was performed according to our pre-registered protocol (CRD42021266888, PROSPERO) with some amendments in the selection and assessment of outcomes.We adopted the concept of the minimum clinically important difference (MCID) [27] to assess the clinical significance of CURs for treating knee OA. The study was conducted by the guidance of the Cochran Handbook for Systematic Review of Interventions [28], and reported according to the Preferred Reporting Items for Systematic Review and Meta-Analysis checking list (Supplementary Table 1) [29].

### Literature search

An electronic literature retrieval was conducted on August 2022. The Cochrane Library, Medline via PubMed, Web of Science, Embase, CNKI (China National Knowledge Infrastructure), SinoMed (Chinese BioMedical Literature Service System), Wanfang and VIP databases, and ClinicalTrials.gov (http://ClinicalTrials.gov) were searched for all published randomized controlled trials (RCTs) evaluating the efficacy and safety of CURs alone in treating knee OA, without time or language restriction. The retrieval strategy sample of PubMed and Embase is shown in Supplementary Table 2.

### Study selection

#### Eligibility criteria

Eligible RCTs were included in this study based on the following criteria: (1) participants: patients diagnosed with knee OA according to the criteria proposed by the American College of Rheumatology (ACR) [30]; (2) intervention: oral CURs; (3) control: oral conventional agents or placebo; (4) one or more of the following outcomes: visual analog scale (VAS) for pain, Western Ontario and McMaster Universities Osteoarthritis Index (WOMAC) total score, WOMAC subscale scores (pain, function and stiffness scores), withdraw rate, concomitant rescue medications, OA biomarkers and adverse events (AEs); and (6) study design: RCTs. Studies were excluded if they met any of the following criteria: (1) studies in which CURs are combined with other treatments; (2) studies lacking essential data; (3) studies in which full-texts were unavailable.

#### Selection process

To select relevant studies for further assessment, two independent reviewers (F.X. and X.M.) removed duplicate publications using Endnote X9, and identified each citation as eligible, ineligible and uncertain by screening titles and abstracts. For eligible and uncertain records, full-texts were further assessed to confirm if the studies were RCTs comparing CURs alone versus conventional therapies or placebo in the treatment of knee OA.

### Data extraction and data items

All data were extracted, and recorded in Excel spreadsheets prepared in advance by two reviewers (L.L.T and Z.L.). The following contents were extracted: (1) study characteristics; (2) patient demographics; and (3) outcomes data. Predefined primary outcomes included VAS for pain, WOMAC pain score, WOMAC function scores, and adverse events. Other outcomes were defined as secondary outcomes. When the data of two or more studies were originated from one clinical trial, only the latest studies providing requisite outcomes were included, and they will be regarded as one study. Attempts were made to obtain missing data by contacting the corresponding author, browsing supplementary files, or consulting relevant data from previous meta-analyses.

### Methodological quality assessment

Two reviewers (L.L.T and Z.L.) applied the recommended Cochran Risk of Bias Tool 1 [31] to assess the risk of bias of the included studies. Each study was judged as having low, unclear, or high risk of bias on the basis of the following assessment domains: random sequence generation (selection bias), allocation concealment (selection bias), blinding of participants and personnel (performance bias), blinding of outcome assessment (detection bias), incomplete outcome data (attrition bias), selective reporting (reporting bias) and other bias.

### Statistical analysis

All data were processed and analyzed by J.F. and Z.L. using the Stata 14 (StataCorp, College Station, Texas, USA) and RevMan 5.4 software (The Cochrane Collaboration, Copenhagen, Denmark). We performed meta-analysis to merge the treatment effects of CURs and control groups, using a random-effects model due to the existence of substantial variability within and between studies [32]. Continuous outcomes were reported as the weighted mean difference (WMD) with 95% confidence interval (CI), and risk ratios (RR) with 95% CI were calculated for dichotomous outcomes. The pooled effect size with a p-value < 0.05 was defined as statistically significant. The MCID, defined as the minimal magnitude an subjective outcome must change to achieve clinical efficacy meeting the satisfaction of patients and clinicians [27], was adopted as a test threshold for clinical significance.. The MCID threshold for the VAS and WOMAC scores was defined as a 20% fluctuation from the baseline of the included studies according to previous researches [33–36], and calculated as follows: 1.18/10 for VAS for pain, 8.97/96 for WOMAC total score, 2.12/20 for WOMAC pain score, 6.62/68 for WOMAC function score, and 0.76/8 for WOMAC stiffness score. Inter-study heterogeneity was assessed by χ2-based Q-test and the I2 index, and an I2 value of 50% was defined as the demarcation of low and high heterogeneity. To explore the influence of various factors on primary pain- and function-related outcomes, we carried out pre-planned subgroup analyses for the placebo-controlled group based on daily dose of CURs (dose < 1,000 mg, or dose ≥ 1,000 mg), total dose of CURs (dose < 50 g, or dose ≥ 50 g), follow-up duration (time < 12 weeks, or time ≥ 12 weeks), type of CURs (bio-optimized or pure extracts) and regions (Asia or non-Asia). Publication bias was detected using funnel plots and Egger’s test for outcomes involving five or more comparisons. The robustness of the quantitative synthesis was tested by omitting the data of each citation in sensitivity analysis. Other outcomes that cannot be merged quantitatively were summarized as narrative reviews.

### Evidence evaluation

The quality of evidence was classified using the GRADE system [37] as high, moderate, low, or very low, with descending assignment of 4, 3, 2, or 1. As the included studies were all RCTs, the level of each outcome began as high quality, but the confidence of each evidence could be decreased by considering the following domains: (1) study limitations; (2) inconsistency of results; (3) indirectness of evidence; (4) imprecision; and (5) publication bias. When evaluating the study limitations [38], the quality of evidence could be downgraded from high level according to results of literature quality assessment, for example, if a study was defined as having unclear risk of bias when it was likely to lower confidence in the estimate of effect size, and the quality of the related outcomes would be decreased by minus 1 to moderate. The I2 index values were used to evaluate the consistency [8, 39] grading: I2 ≤ 50% equalled ‘not serious’ quality downgrade; 50% < I2 ≤ 75% equalled ‘serious’ quality downgrade (minus 1); I2 > 75% equalled ‘very serious’ quality downgrade (minus 2). We applied the MCID in grading imprecision for VAS and WOMAC scores on the premise that the results were not statistically significant: the 95% CI exceeded the MCID either in the upper or lower confidence limit equalled ‘serious’ quality downgrade (minus 1); the 95% CI of WMD encompassed the MCID equalled “very serious” quality downgrade (minus 2). The assessment of imprecision [40] for RR was implemented by strictly adhering to the GRADE guidelines. As indirectness was appraised by the stringent inclusion and exclusion criteria, reassessment was not necessary. Publication bias was evaluated according to the results of funnel plots and Egger’s test.

## Results

### Literature search results

**Fig. 1:**
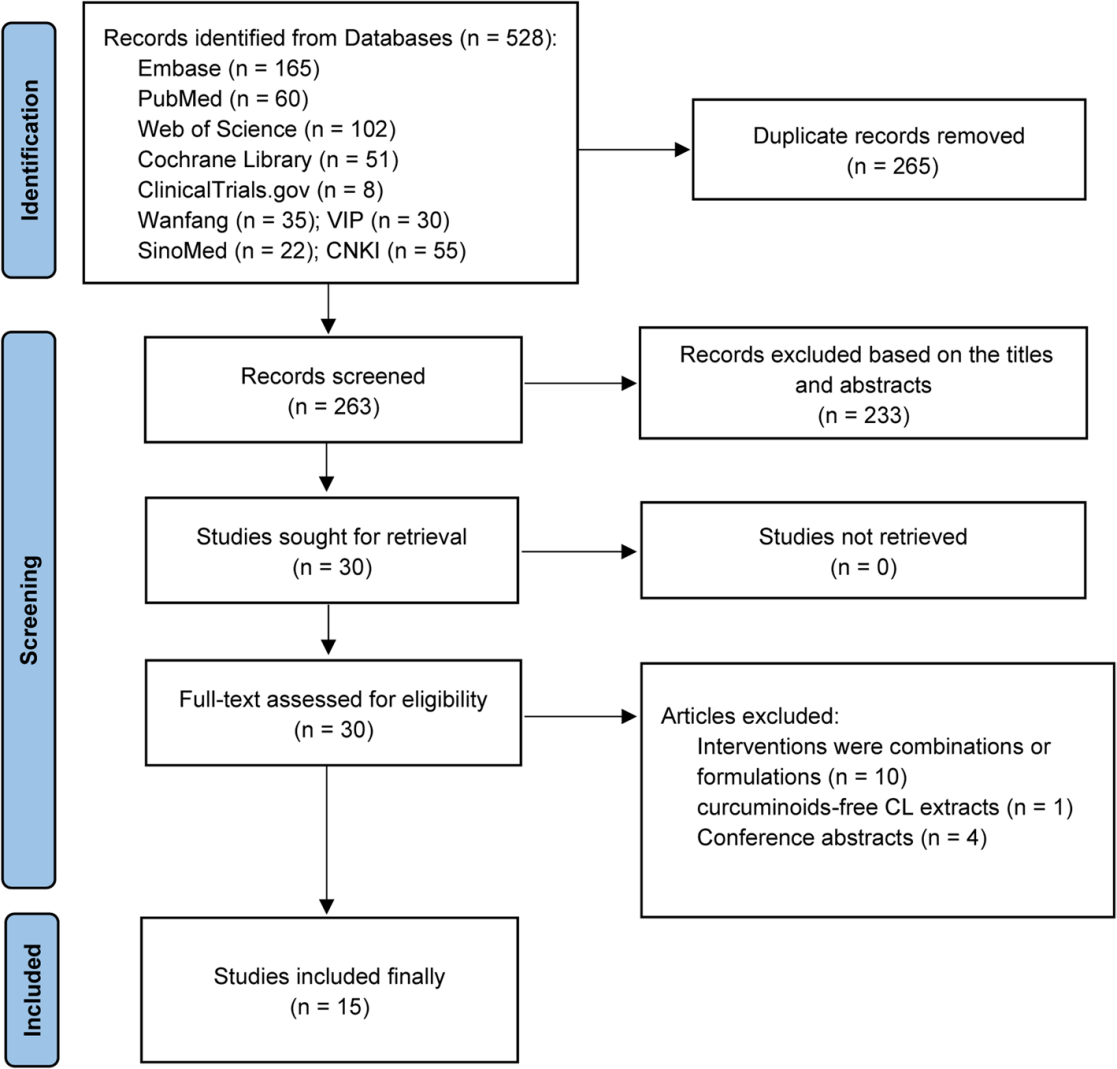
Flow chart of literature screening

The literature screening process is illustrated in Fig. 1. Initial literature retrievals obtained  528 citations, 265 of which were removed for duplicate publications. After screening the titles and abstracts of the remaining  263 citations, we excluded  233 irrelevant records based on the selection criteria. Thirty studies were remained for full-text assessment, eleven studies were excluded because of ineligible interventions, including the combination of glucosamine hydrochloride, chondroitin sulphate and curcumin [41], curcumagalactomannoside complex [42, 43], CURs combined with diclofenac [44, 45], herbal formulations of different extracts [46–50], and CURs-free CL extracts [51]. Furthermore, we excluded four conference abstracts [52–55] due to incomplete data. Finally,  fifteen eligible studies with 1670 patients were enrolled in our analysis 531.

#### Basic characteristics of studies

The study characteristics are presented in Table 1. All included studies were CURs-intervened trials aimed to evaluate the clinical effectiveness of CURs, and published between 2009 and 2022. Sample sizes of included studies were ranged from 30 to 331, and the follow-up durations were limited in 6 months. The details of CURs preparations and administration protocols are presented in Tables 2 and 3. Five trials [56–60] applied active-controlled arms (ibuprofen, diclofenac, and paracetamol), and the other ten [61–72] were all placebo-controlled trials.

**Table 1:** The basic characteristic of included studies

Study	Region	Outcomes	Treatment		Sample size (female/male)		Age (years)		K–L grade	Follow-up duration	Funder
			**Intervention**	**Control**	**Intervention**	**Control**	**Intervention**	**Control**			
Kuptniratsaikul V 2009 [56]	Thailand	VAS, AEs	CL extract	Ibuprofen	52 (41/11)	55 (45/10)	61.4 ± 8.7	60.0 ± 8.4	-	6 weeks	Research department
Kuptniratsaikul V 2014 [57]	Thailand	WOMAC, AEs	CL extract	Ibuprofen	171 (157/14)	160 (139/21)	60.30 ± 6.8	60.9 ± 6.9	1–4	4 weeks	Research department
Nakagawa Y 2014 [61]	Japan	VAS, AEs, Inflammatory Biomarkers	Nano-curcumin (Theracurmin®)	Placebo	18 (14/4)	23 (18/5)	66.10 ± 7.2	71.9 ± 5.3	2–3	8 weeks	Private corporation
Panahi Y 2014 [62], Panahi Y 2015 [63], Rahimnia AR 2015 [64]	Iran	VAS, WOMAC, AEs, Inflammatory Biomarkers	Curcuminoids (C3 complex®)	Placebo	19 (14/5)	21 (17/4)	57.32 ± 8.78	57.57 ± 9.05	-	6 weeks	Research department
Srivastava S 2016 [65]	India	VAS, WOMAC, AEs, Inflammatory Biomarkers	CL extract (Haridra®)	Placebo	78 (53/25)	82 (50/32)	50.23 ± 8.08	50.27 ± 8.63	1–4	4 months	Research department
Haroyan A 2018 [66]	Armenia	WOAMC, AEs, laboratory indicators	Curcuminoids (CuraMed®)	Placebo	66 (60/6)	68 (65/3)	54.65 ± 8.84	56.04 ± 8.55	1–3	12 weeks	Private corporation
Panda SK 2018 [67]	India	VAS, WOMAC, AEs, Inflammatory Biomarkers	Curcuminoids (Curene®)	Placebo	25	25	55.2 ± 8.58	53.12 ± 8.25	2–3	2 months	Private corporation
Gupte PA 2019 [58]	India	VAS, AEs, Inflammatory Biomarkers	Curcumin (Longvida®)	Ibuprofen	17 (11/6)	25 (23/2)	57 ± 7.5	54.0 ± 8.0	1–4	3 months	Private corporation
Herotin Y 2019 [68]	Belgium	VAS, AEs, Inflammatory Biomarkers	Curcumin (FLEXOFYTOL®)	Placebo	47 (40/7) 49 (39/15)	45 (34/11)	61.4 ± 7.49 60.9 ± 9.78	63.3 ± 7.69	2–4	6 months	Private corporation
Shep D 2019 [59]	India	VAS, AEs, Inflammatory Biomarkers	Curcuminoids (BCM-95®)	Diclofenac	70 (25/45)	69 (21/48)	53.09 ± 4.17	52.14 ± 3.76	-	4 weeks	Not applicable
Wang Z 2020 [69]	Australia	VAS, WOMAC, AEs, MRI	CL extract (Turmacin Plus)	Placebo	36 (18/18)	34 (21/13)	61.3 ± 8.5	62.4 ± 8.8	-	12 weeks	Private corporation
Atabaki M 2020 [70]	Iran	VAS, AEs, Inflammatory Biomarkers	Nano-curcumin (SinaCurcumin®)	Placebo	15 (15/0)	15 (15/0)	49.13 ± 5.81	48.26 ± 5.11	2–3	3 months	Research department
Hashemzadeh K 2020 [71]	Iran	WOMAC, AEs, Inflammatory Biomarkers	Nano-curcumin (SinaCurcumin®)	Placebo	36 (29/7)	35 (31/4)	54.11 ± 5.8	56.54 ± 5.77	2–3	6 weeks	Research department
Singhal S 2021 [60]	India	WOAMC, AEs	Curcuminoids (BCM-95®)	Paracetamol	73 (53/20)	71 (54/17)	53.1 ± 10.9	50.8 ± 9.9	2–3	6 weeks	Not applicable
Lopresti L 2022 [72]	Australia	VAS, AEs	Curcumin (Curcuge®)	Placebo	51 (24/27)	50 (26/24)	59.6 ± 6.57	57.9 ± 6.22	-	8 weeks	Private corporation

*CL* Curcuma longa L, *VAS* visual analog scale, *WOMAC* Western Ontario and McMaster Universities osteoarthritis index, *AEs* adverse events, *MRI* magnetic resonance imaging, *K–L* Kellgren–Lawrence, *BMI* body mass index

**Table 2:** Characteristics of curcuminoids formulations applied in the included studies

Studies	Formulations	Source	Compounds or species, concentration	Purity or encapsulation rate (%)	Quality control reported? (Y/N)	Chemical analysis reported? (Y/N)
Kuptniratsaikul V 2009 [56]	CL extract	Thai Government Pharmaceutical Organization	Curcuminoids extracted from the dried rhizomes of CL, 250 mg per capsule	Not mention	Y – Prepared according to the Good Manufacturing Procedures Standard	N
Kuptniratsaikul V 2014 [57]	CL extract	Thai Government Pharmaceutical Organization	Curcuminoids extracted from the dried rhizomes of CL, 250 mg per capsule	75% – 85%	Y – Prepared according to the Good Manufacturing Procedures Standard	Y – HPLC
Nakagawa Y 2014 [61]	Theracurmin®	Theravalues, Tokyo, Japan	Surface-controlled water-dispersible nano-curcumin, 30 mg per capsule	Not mentioned	N	N
Panahi Y 2014 et al. [62]	C3 complex®	Sami Labs Ltd., Bangalore, India	Curcuminoids and bioperine, 500 mg and 5 mg per capsule	Not mentioned	N	N
Srivastava S 2016 [65]	Haridra®	Himalaya Drug Company Bangalore, India	Curcuminoids extracted from rhizomes of CL, 500 mg per capsule	≥ 95%	N	Y – HPTLC
Haroyan A 2018 [66]	CuraMed®	EuroPharma, USA	Curcuminoids and volatile oil extracted from rhizomes of CL, 500 mg and 49–52 mg per capsule	Not mentioned	Y – Prepared according to the Good Manufacturing Practice	N
Panda SK 2018 [67]	Curene®	Olene Life Sciences Pvt. Ltd., Chennai, India	Curcuminoids and volatile oil extracted from rhizomes of CL, 500 mg per capsule	≥ 95%	Y– Developed using proprietary technology called Aqueosome®	N
Gupte PA 2019 [58]	Longvida®	Verdure Sciences, USA	Solid lipid curcumin particles containing 400 mg patented lipophilic matrix and 80 mg curcumin per capsule	Not mentioned	Y – Made with SLCP™ Technology	N
Herotin Y 2019 [68]	FLEXOFYTOL®	Tilman SA, Baillonville, Belgium	Curcuminoids extracted from extracted from rhizomes of CL, polysorbate 80 [E433]as an emulsifier, and citric acid [E330] as an acidity regulator, 46.67 mg per capsule	Pharmaceutical-grade	N	N
Shep D 2019 [59]	BCM-95®	Arjuna Natural Ltd., Kerala, India	Curcuminoids and volatile oil extracted from rhizomes of CL, 500 mg per capsule	≥ 95%	N	N
Wang Z 2020 [69]	Turmacin Plus	Natural Remedies Pvt., Bengaluru	Curcuminoids and turmerosaccharides extracted from rhizomes of CL, 500 mg per capsule	20%	N	N
Atabaki M 2020 [70]	SinaCurcumin®	Exir Nano Sina Company, Tehran, Iran	Nanomicelle consists of curcumin, 80 mg per capsule	≈ 100%	N	N
Hashemzadeh K 2020 [71]	SinaCurcumin®	Exir Nano Sina Company, Tehran, Iran	Nanomicelle consists of curcumin, 80 mg per capsule	≈ 100%	N	N
Singhal S 2021 [60]	BCM-95®	Arjuna Natural Pvt. Ltd., India	Curcuminoids and volatile oil extracted from rhizomes of CL, 500 mg per capsule	≥ 95%	N	Y – UPLC, FT-NIR spectrometer
Lopresti L 2022 [72]	Curcuge®	Dolcas Biotech LLC	Standardised curcuminoids extract, 500 mg per capsule	≈ 50%	N	N

*CL* Curcuma longa L., *HPLC* high-performance liquid chromatography, *HPTLC* high-performance thin layer chromatography, *UPLC* ultra-performance liquid chromatography, *FT-NIR spectrometer*, Fourier transform near infrared spectrometer

**Table 3:** Administration protocols of curcuminoids and rescue medications for patients of included studies

**Studies**	**Dose (mg/time)**	**Frequency (time/day)**	**Daily dose** ^a^ **(mg/day)**	**Total dose** ^b^ **(g)**	**Rescue medications**
Kuptniratsaikul V 2009 [56]	500	4	2000	84	
Kuptniratsaikul V 2014 [57]	500	3	1500	42	Tramadol
Nakagawa Y 2014 [61]	90	2	180	10.08	Celecoxib
Panahi Y 2014 et al. [62]	500	3	1500	63	Naproxen
Srivastava S 2016 [65]	500	1	500	60	Diclofenac
Haroyan A 2018 [66]	500	3	1500	126	
Gupte PA 2019 [67]	80	2	160	14.4	
Panda SK 2018 [58]	500	1	500	30	Paracetamol
Herotin Y 2019 a [68]	93.34	2	186.68	33.6	Paracetamol / NSAIDs
Herotin Y 2019 b [68]	140.01	2	280.02	50.4	
Shep D 2019 [59]	500	3	1500	42	Paracetamol
Wang Z 2020 [69]	500	2	1000	84	Paracetamol
Atabaki M 2020 [70]	80	1	80	7.2	Diclofenac
Hashemzadeh K 2020 [71]	40	2	80	3.36	
Singhal S 2021 [60]	500	2	1000	42	
Lopresti L 2022 [72]	500	2	1000	56	

^a^ The daily dose of curcuminoids is equal to the product of the dose and frequency

^b^ The total dose of curcuminoids is equal to the product of the daily dose and follow-up duration

#### Quality assessment

**Fig. 2:**
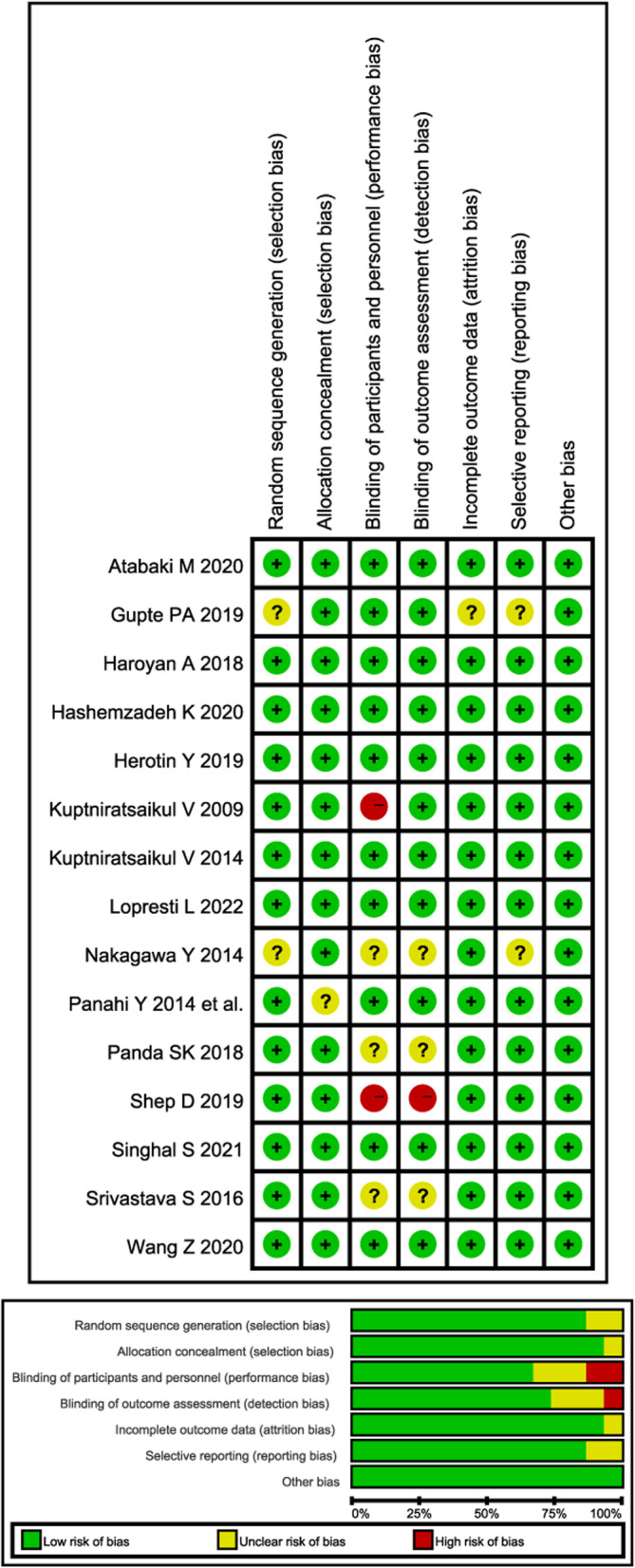
Risk of bias summary

The results of literature quality assessment based on the Cochran Risk of Bias Tool 1 are presented in Fig. 2. Overall, eight studies [56, 60, 66, 68–72] were defined as having low risk of bias, and five studies [58, 61, 62, 65, 67] were judged as having moderate risk of bias for potential reporting bias [61] and attrition bias [67], and deficiencies in specific descriptions of randomization [58, 61], allocation concealment [62] or blinding methods [61, 65, 67]. Two studies [56, 59] were judged as having high risk of bias for inadequate procedures in blind methods. An appropriate description of random sequence generation was reported in thirteen studies [56, 57, 59, 60, 62, 65–72], reasonable allocation concealment was performed in fourteen studies [56–61, 65–72], and double-blinded methods were specific in ten studies [57, 60, 62, 66–72].

#### VAS for pain

**Fig. 3:**
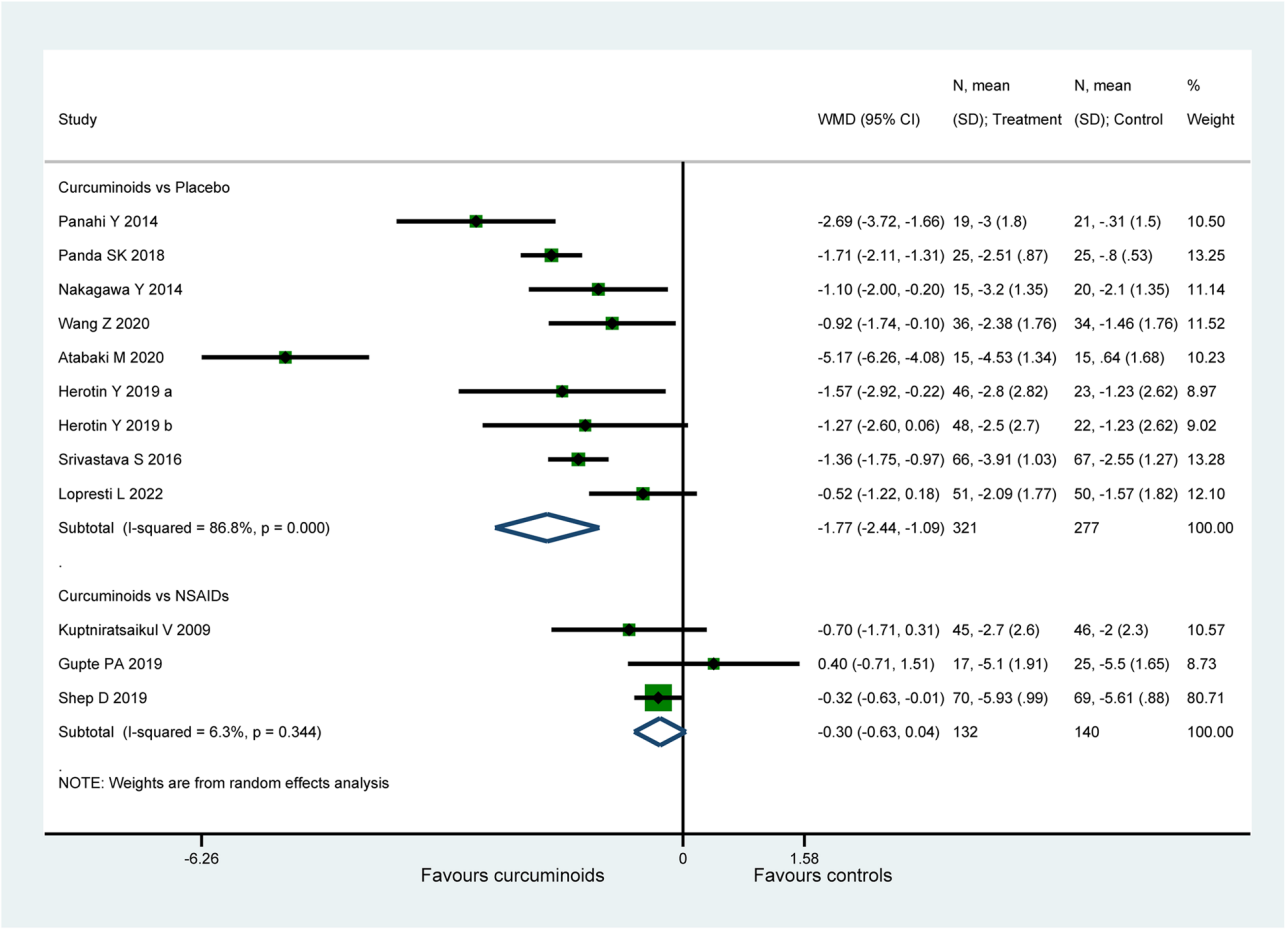
Forest plot portraying the weighted mean difference with 95% confidence interval of VAS for pain

Eleven studies ( 870 patients) [56, 58, 59, 61, 62, 65, 67–70, 72] assessed knee pain using VAS for pain. When compared to placebo, CURs were found to be more efficacious on the improvement of VAS for pain ( WMD: − 1.77, 95% CI: − 2.44 to − 1.09, P < 0.001, I2 = 86.8%, Fig. 3). Whereas there was no significant difference detected between CURs and NSAIDs (WMD: − 0.3, 95% CI: − 0.63 to 0.04, *P* = 0.082, I2 = 6.3%, Fig. 3). For the comparison between CUR and NSAIDs, the therapeutic effect (− 0.3) was smaller than the MCID (1.18 for VAS for pain). However, the therapeutic effect (− 1.77) of CURs in placebo-controlled group exceeded the MCID with both statistical and clinical significance.

An obvious decrease from 86.8% to 56.9% in heterogeneity for placebo-controlled group was observed after removing the study of Atabaki et al. [70], with the intervention of CURs loaded nano-micelles, the pooled result ( WMD: − 1.36, 95% CI: − 1.76 to − 0.97, P < 0.001) was similar with the original analysis. Sensitivity analysis did not ferret out one individual study that would affect the statistical robustness of the overall results.

#### WOMAC total score

**Fig. 4:**
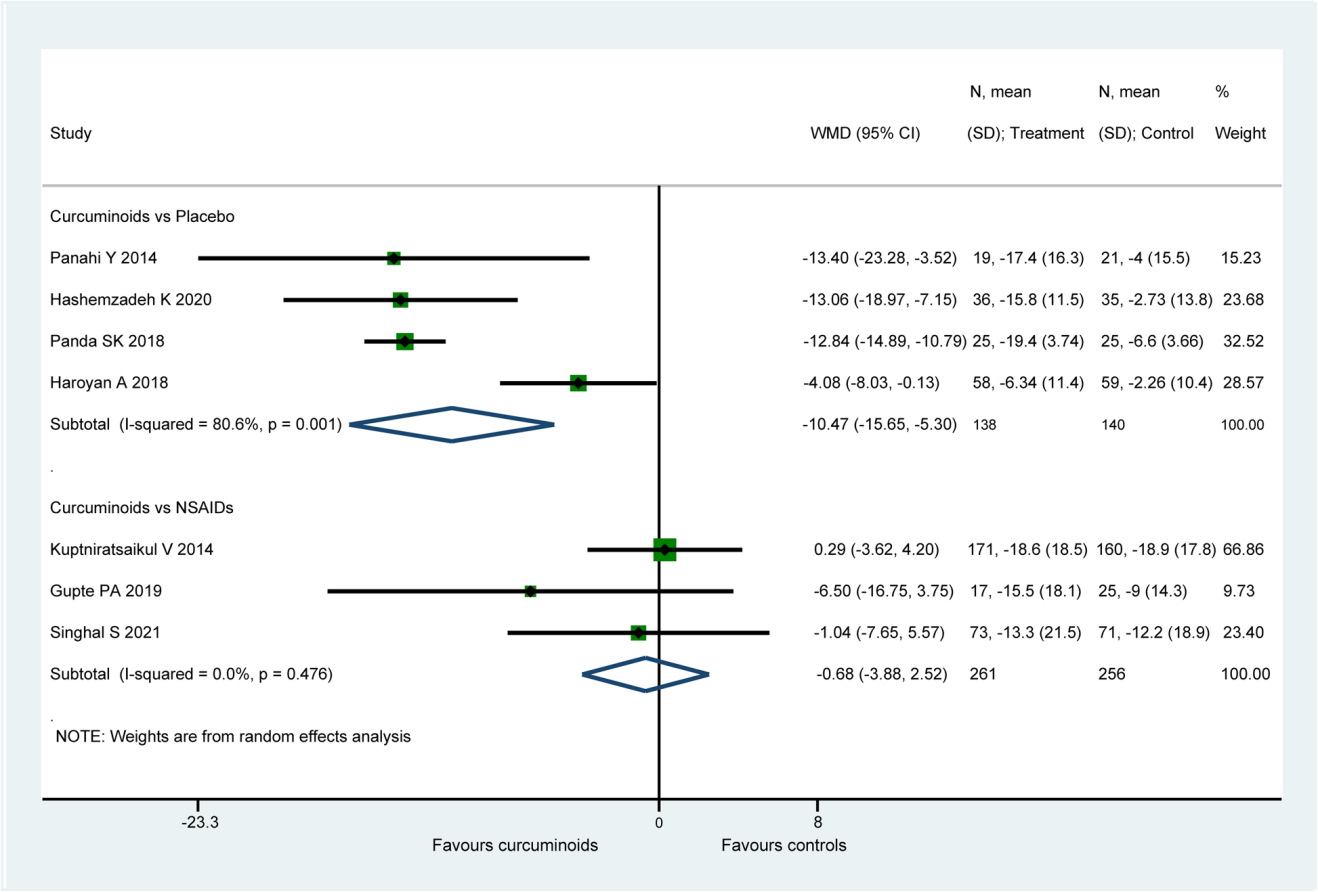
Forest plot portraying the weighted mean difference with 95% confidence interval of WOMAC total score

Seven study (795 patients) [57, 58, 60, 62, 66, 67, 71] reported the data of WOMAC total score. When compared to placebo, CURs were found to be more efficacious on the improvement of WOMAC total score (WMD: − 10.47, 95% CI: − 15.65 to − 5.3, *P* < 0.001, I2 = 0.0%, Fig. 4). Whereas there was no significant difference found between CURs and NSAIDs (WMD: − 0.68, 95% CI: − 3.88 to 2.52, *P* = 0.676, I2 = 80.6%, Fig. 4). For the comparison between CURs and NSAIDs, the therapeutic effect (− 0.68) did not exceed the MCID (8.97 for WOMAC total score), while the effect size (− 10.47) of CURs in placebo-controlled group was larger than the MCID with both statistical and clinical significance.

When the data of Haroyan et al. [66] was omitted, a significant reduction in heterogeneity from 80.6% to 0.0% in placebo-controlled group was observed, but the pooled result (WMD =  − 12.88, 95% CI: − 14.79 to − 10.98, P < 0.001) of remained studies was similar with the original analysis. Sensitivity analysis did not ferret out one individual study that would affect the statistical robustness of the overall results.

#### WOMAC pain score

**Fig. 5:**
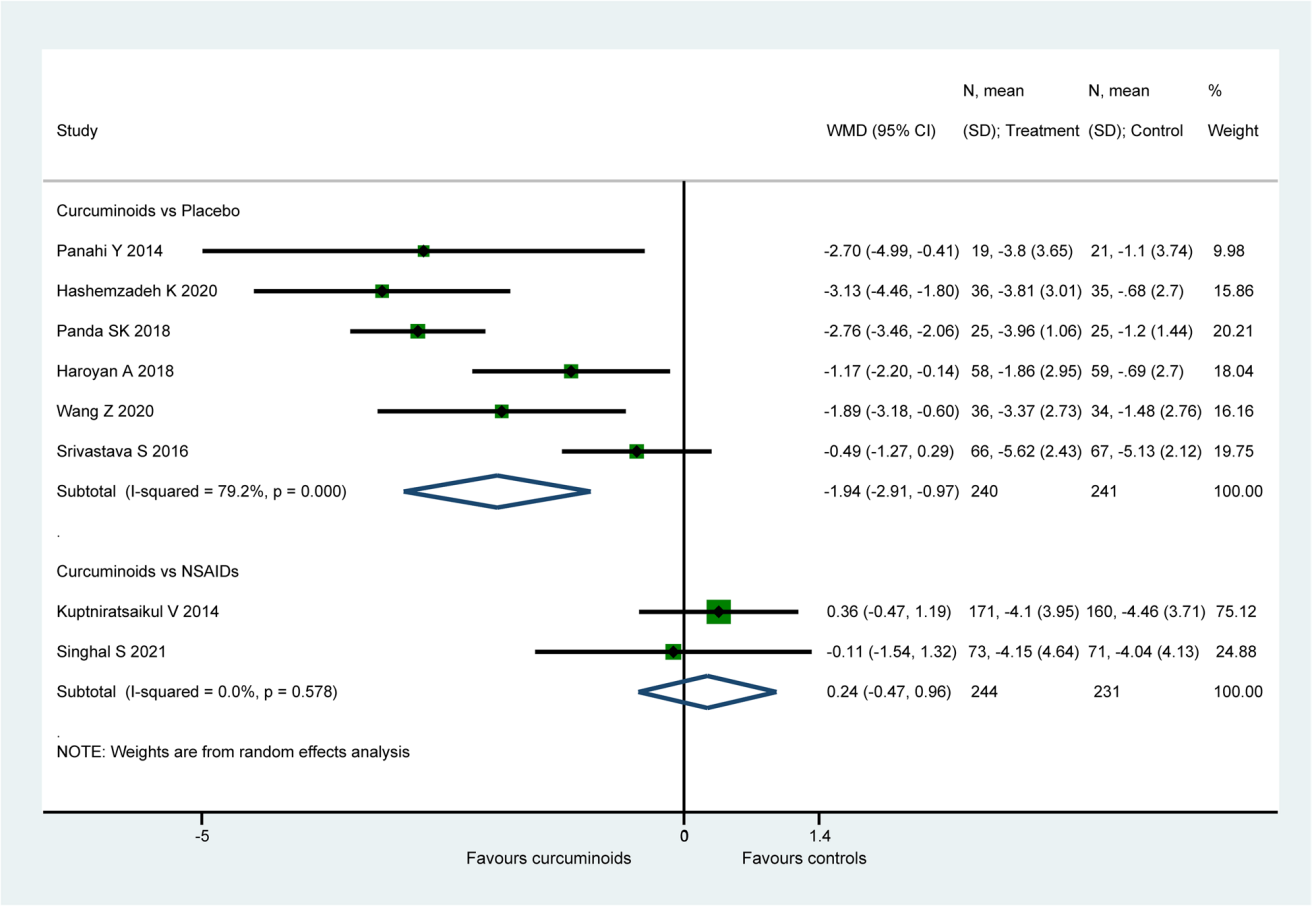
Forest plot portraying the weighted mean difference with 95% confidence interval of WOMAC pain score

Eight studies (956 patients) [57, 60, 62, 65–67, 69, 71] reported the data of WOMAC pain score. When compared to placebo, CURs were found to be significantly more efficacious on the improvement of WOMAC pain score (WMD: − 1.94, 95% CI: − 2.91 to − 0.97, *P* < 0.001, I2 = 79.2%, Fig. 5). There is no significant difference detected between CURs and NSAIDs (WMD: 0.24, 95% CI: − 0.47 to 0.96, *P* = 0.505, I2 = 0.0%, Fig. 5). For the comparison between CURs and NSAIDs, the therapeutic effect (0.24) did not exceed the MCID (2.12 for WOMAC pain score). Similarly, the effect size (− 1.94) of CURs in placebo-controlled group was smaller than the MCID with only statistical significance.

The study of Srivastava et al. [65] was considered to be the potential source of heterogeneity given that the I2 values in placebo-controlled group decreased from 79.2% to 51.8% after omitting their data, and the pooled result (WMD: − 2.28, 95% CI: − 3.05 to − 1.52, P < 0.001) reached up to the magnitude exceeding the threshold (2.12) for clinical significance. Sensitivity analysis did not ferret out one individual study that would affect the statistical robustness of the overall results.

#### WOMAC function score

**Fig. 6:**
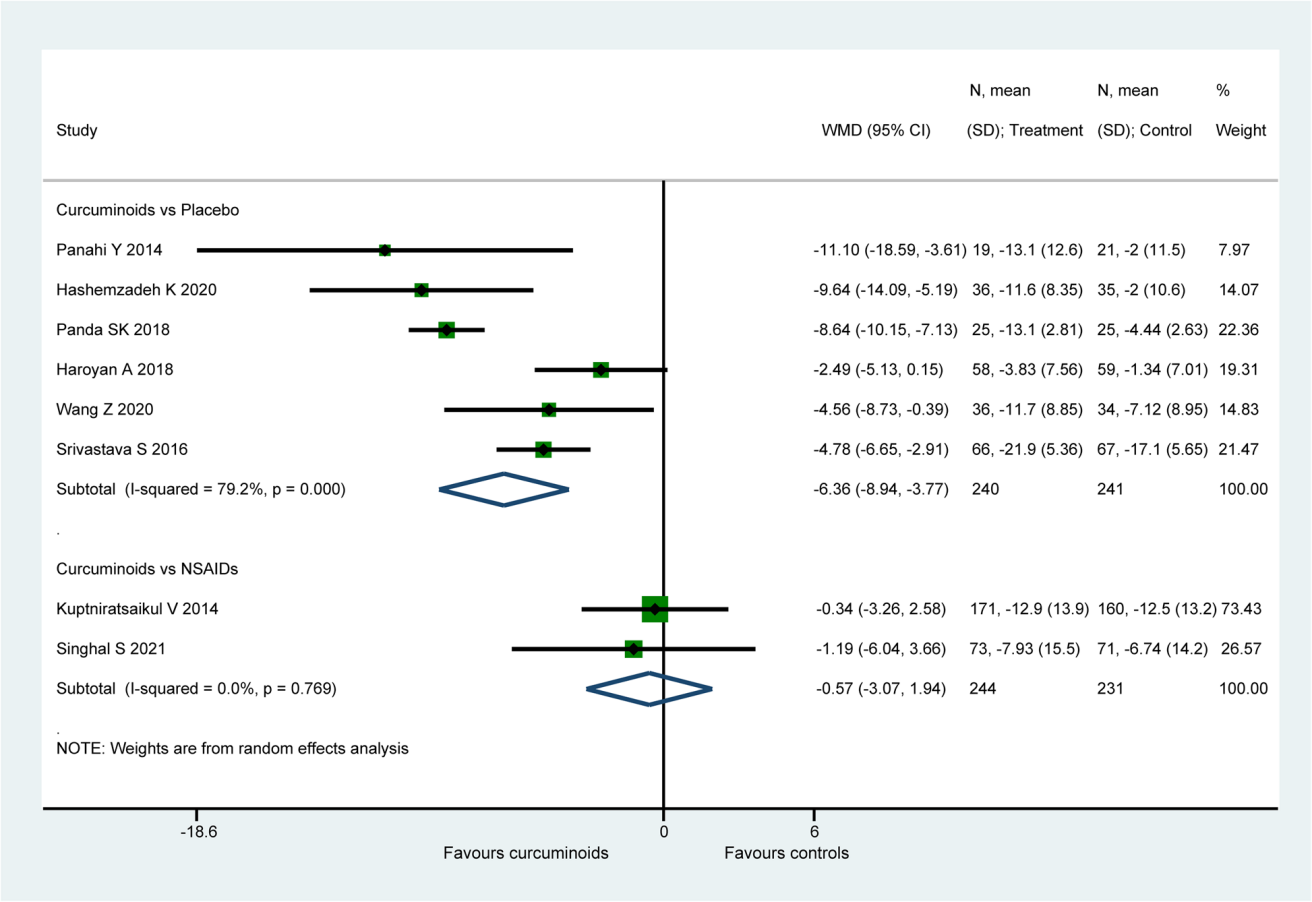
Forest plot portraying the weighted mean difference with 95% confidence interval of WOMAC function score

Eight studies (956 patients) [57, 60, 62, 65–67, 69, 71] assessed joint function using the WOMAC function score. When comparing to placebo, CURs were found to be significantly more efficacious on the improvement of WOMAC function score (WMD: − 6.36, 95% CI: − 8.94 to − 3.78, *P* < 0.001, Fig. 6). However, there is no significant difference found between CURs and NSAIDs (WMD: − 0.57, 95% CI: − 3.07 to 1.94, *P* = 0.657, I2 = 0.0%, Fig. 6). For the comparison between CURs and NSAIDs, the therapeutic effect (− 0.57) did not exceed the MCID (6.62 for WOMAC function score). Similarly, the effect size (− 6.36) of CURs in placebo-controlled group was smaller than the MCID with only statistical significance.

After excluding the study of Haroyan et al. [66], the inter-study heterogeneity in placebo-controlled group slightly decreased from 79.2% to 70.7%, but the pooled result (WMD: − 7.21, 95% CI: − 9.71 to − 4.72, *P* < 0.001) reached up to the magnitude exceeding the threshold (6.62) for clinical significance. Sensitivity analysis did not ferret out one individual study that would affect the statistical robustness of the overall results.

#### WOMAC stiffness score

**Fig. 7:**
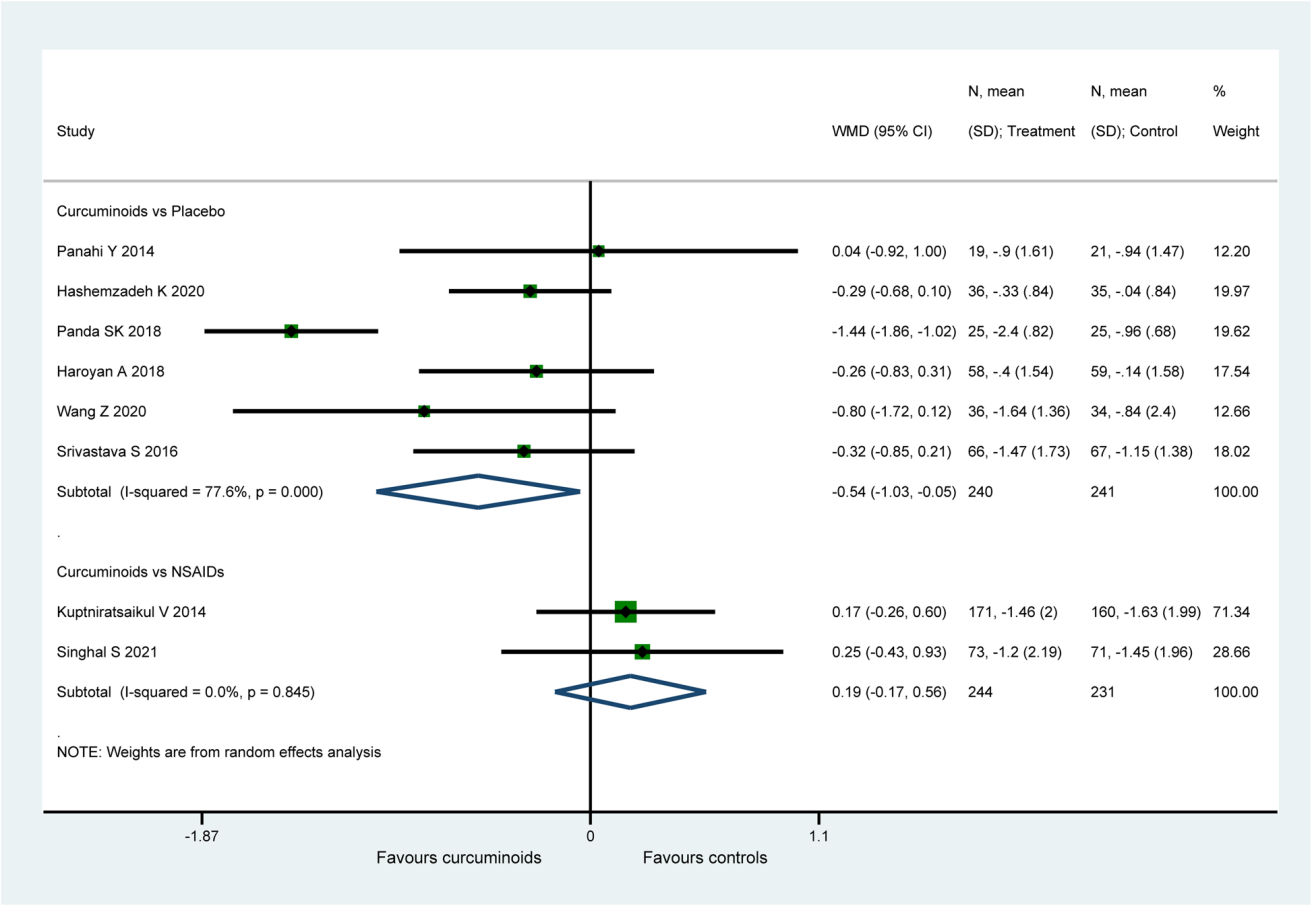
Forest plot portraying the weighted mean difference with 95% confidence interval of WOMAC stiffness score

Eight studies (956 patients) [57, 60, 62, 65–67, 69, 71] evaluated joint stiffness status using the WOMAC stiffness score. CURs were found to be significantly more efficacious on the improvement of WOMAC stiffness score (WMD: − 0.54, 95% CI: − 1.03 to − 0.05, *P* = 0.031, I2 = 77.6%, Fig. 7) when compared with placebo. There is no significant difference found between CURs and NSAIDs (WMD: 0.19, 95% CI: − 0.17 to 0.56, *P* = 0.298, I2 = 0.0%, Fig. 7). For the comparison between CURs and NSAIDs, the therapeutic effect (0.19) did not exceed the MCID (0.76 for WOMAC stiffness score). Similarly, the effect size (− 0.54) of CURs in placebo-controlled group was smaller than the MCID with only statistical significance.

By excluding the study of Panda et al. [67], a decrease in heterogeneity from 77.6% to 0.0% in placebo-controlled group was observed, but the pooled result (WMD: − 0.31, 95% CI: − 0.56 to − 0.05, *P* = 0.018) was similar with the original analysis. Sensitivity analysis did not ferret out one individual study that would affect the statistical robustness of the overall results.

### Adverse events

**Fig. 8:**
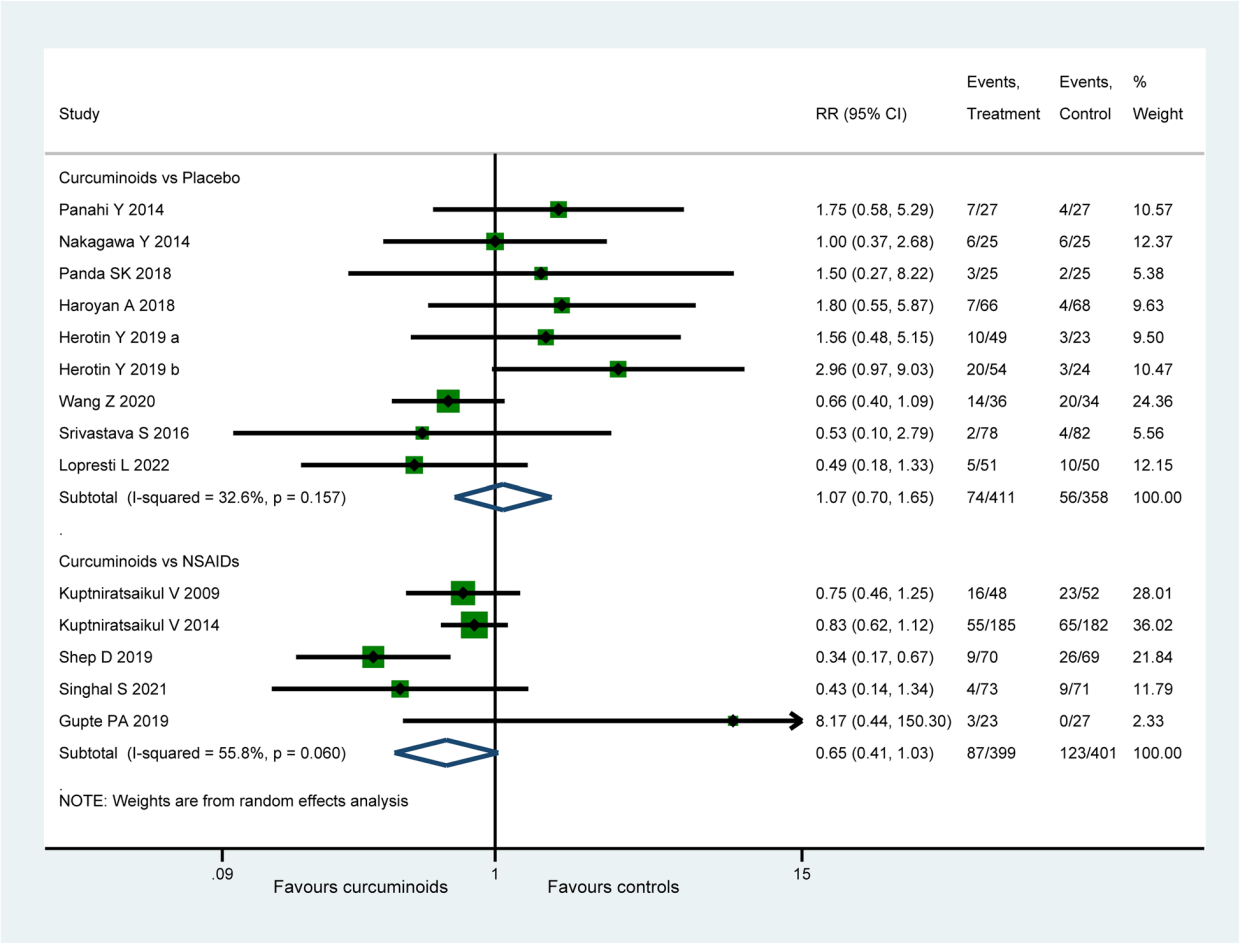
Forest plot portraying the risk ratio with 95% confidence interval of adverse events

Among the included fifteen studies, two [70, 71] reported no AEs at the end of the trials. According to the data of the remaining thirteen studies ( 1569 patients), AEs were mainly concentrated in gastrointestinal symptoms including meteorism, gastro-oesophageal reflux, dyspepsia, nausea, and stomach pain as shown in Table 4. There was no significant difference found between CURs and placebo group in the incidence of AEs ( RR: 1.07, 95% CI: 0.70 to 1.65, *P* = 0.745, I2 = 32.6%, Fig. 8), while a lower incidence of AEs was observed in CURs group when compared with NSAIDs group, but the pooled results were not statistically significant ( RR: 0.65, 95% CI: 0.41 to 1.03, *P* = 0.065, I2 = 55.8%, Fig. 8). Sensitivity analysis found that the difference between CURs and NSAIDs groups became statistically significant (RR: O.63, 95% CI: 0.41 to 0.95, *P* = 0.026, I2 = 53.7%) when the data of Gupte et al. [58] were omitted.

**Table 4:** The incidence of adverse events in curcuminoids and control groups

Groups	Curcuminoids group		Control group	
Adverse events	**Total (n)**	**Incidence (%)**	**Total (n)**	**Incidence (%)**
Dyspepsia	34	4.20	52	6,85
Nausea	24	2.96	27	3.56
Stomach pain	38	4.69	62	8.17
Diarrhea and/or constipation	47	5.80	40	5.27
Meteorism and gastro-esophageal reflux	21	2.59	31	4.08
Impairment of liver and/or kidney function	1	0.12	1	0.13
Others	38	4.69	57	7.51
Summary	203	25.06	270	35.57

### OA biomarkers

Two studies [62, 65] assessed the antioxidation of CURs through detecting the serum level of reactive oxygen species (ROS), superoxide dismutase (SOD), glutathione (GSH) and malondialdehyde (MDA), and found that changes in these biomarkers may contribute to the therapeutic effects of CURs in alleviating OA symptoms. Three studies [58, 64, 65] reported the serum level of inflammatory mediators, such as interleukin-1β (IL-1β), IL-4, IL-6, tumor necrosis factor-α (TNF-α), leukotriene B4 (LTB4) and prostaglandin E2 (PGE2), and proved that the systemic anti-inflammatory effects of CURs may have no correlation with its therapeutic effects in knee OA. Besides, six studies [58, 59, 64, 66, 67, 70] measured the C-reactive protein (CRP) serum concentration and erythrocyte sedimentation rate (ESR), two sensitive biomarkers for systemic inflammation. Two studies [58, 68] evaluated the status of cartilage degeneration via serum level of C-terminal telopeptides of type II Collagen (U-CTX-II) and Coll2-1. Similarly, there was no significant difference found between groups for aforementioned biomarkers.

### Withdraw rate and rescue medications

All included studies reported the withdraw rate of follow-up cohort, there was no lost case reported in the treatment and control group of the study of Atabaki et al. [70], thus their data cannot be merged in meta-analysis. The pooled analysis showed no significant difference in withdraw rate between CURs and placebo group ( RR: 1.02, 95% CI: 0.69 to 1.52, *P* = 0.903, I2 = 0.0%) or NSAIDs group ( RR: 0.87, 95% CI: 0.6 to 1.27, *P* = 0.468, I2 = 14.2%, Supplementary Fig. 1). Eight studies [57, 59, 61, 62, 67–69, 71] reported the administration of concomitant rescue medications for ethical concerns. Among which, five studies [57–59, 67, 68] reported the number of patients using rescue medications, the pooled analysis found no significant difference in the usage of rescue medications between CURs and placebo group (RR: 0.93, 95% CI: 0.6 to 1.43, *P* = 0.742, I2 = 55.4%) or NSAIDs group (RR: 0.99, 95% CI: 0.53 to 1.83, *P* = 0.963, I2 = 38.8%, Supplementary Fig. 2). Four studies [61, 62, 69, 71] recorded the discontinuation of rescue medications, the pooled results showed that the cessation rate of rescue medications in CURs group was significantly higher than placebo group (RR: 4.04, 95% CI: 2.43 to 6.71, P < 0.001, I2 = 11.7%, Supplementary Fig. 2).

### Subgroup analysis

Subgroup analyses were only performed in the placebo-controlled group due to limited number of original studies, and to avoid the interference of different controls to the results. The results of subgroup analyses are arranged in Table 5. We found no significant difference in the subgroup results of VAS for pain, WOMAC pain score and WOMAC function score compared to the overall analyses, except for the pure extracts subgroup; the result of the subgroup showed no significant difference between CURs and placebo on the improvement of WOMAC pain score. As for clinical significance, the effect size of the pure extracts and non-Asia subgroups decreased to be lower than the MCID for VAS for pain. Conversely, in the time < 12 weeks, daily dose < 1,000 mg, total dose < 50 g, bio-optimized extracts, and Asia subgroups, we found that the effect sizes increased to exceed the MCID for WOMAC pain score and WOMAC function score.

**Table 5:** Subgroup analyses of VAS for pain, WOMAC pian score and WOMAC function score in curcuminoids versus placebo group

Outcomes	VAS for pain			WOMAC pain score			WOMAC function score		
**Subgroups**	**Studies**	**WMD (95% CI)**	**I**^**2**^** (*p*-value)**	**Studies**	**WMD (95% CI)**	**I**^**2**^** (*p*-value)**	**Studies**	**WMD (95% CI)**	**I**^**2**^** (*p*-value)**
**Overall analysis**	8	− 1.94 (− 2.65, − 1.22)	86.3% (*p* < 0.001)	6	− 1.94 (− 2.91, − 0.97)	79.2% (*p* < 0.001)	6	− 6.36 (− 8.94, − 3.77)	79.2% (*p* < 0.001)
**Daily dose of curcuminoids**									
Dose < 1,000 mg	4	− 2.16 (− 3.45, − 0.88)	89.9% (*p* < 0.001)	2	− 2.84 (− 3.46, − 2.22) ^c^	0.0% (*p* = 0.629)	2	− 8.74 (− 10.17, − 7.31) ^d^	86.2% (*p* < 0.001)
Dose ≥ 1,000 mg	4	− 1.31 (− 2.02, − 0.59)	76.1% (*p* = 0.006)	4	− 1.25 (− 2.06, − 0.43)	47.2% (*p* = 0.128)	4	− 4.47 (− 6.63, − 2.31)	55.3% (*p* = 0.135)
**Total dose of curcuminoids**									
Dose < 50 g	4	− 2.37 (− 3.91, − 0.83)	92.3% (*p* < 0.001)	2	− 2.84 (− 3.46, − 2.22) ^c^	0.0% (*p* = 0.629)	2	− 8.74 (− 10.17, − 7.31) ^d^	0.0% (*p* = 0.667)
Dose ≥ 50 g	5	− 1.29 (− 1.91, − 0.68)	68.1% (*p* = 0.014)	4	− 1.25 (− 2.06, − 0.43)	47.2% (*p* = 0.128)	4	− 4.47 (− 6.63, − 2.31)	42.7% (*p* = 0.155)
**Follow-up duration**									
Time < 12 weeks	4	− 1.47 (− 2.25, − 0.68)	79.2% (p = 0.002)	3	− 2.83 (− 3.43, − 2.23) ^c^	0.0% (*p* = 0.884)	3	− 8.83 (− 10.23, − 7.24) ^d^	0.0% (*p* = 0.763)
Time ≥ 12 weeks	4	− 2.04 (− 3.38, − 0.71)	91.2% (*p* < 0.001)	3	− 1.05 (− 1.83, − 0.28)	44.1% (*p* = 0.167)	3	− 4.08 (− 5.51, − 2.65)	0.0% (*p* = 0.372)
**Biological optimization**									
Bio-optimized extracts	5	− 2.25 (− 3.33, − 1.17)	88.0% (*p* < 0.001)	4	− 2.38 (− 3.33, − 1.43) ^c^	61.4% (*p* = 0.051)	4	− 7.45 (− 11.40, − 3.51) ^d^	83.1% (*p* = 0.001)
Pure extracts	3	− 1.00 (− 1.54, − 0.46) ^b^	55.4% (*p* = 0.106)	2	− 1.09 (− 2.45, 0.27) ^a^	70.0% (*p* = 0.068)	2	− 4.74 (− 6.45, − 3.04)	0.0% (*p* = 0.925)
**Regions**									
Asia	5	− 2.32 (− 3.29, − 1.34)	91.5% (*p* < 0.001)	4	− 2.19 (− 3.65, − 0.74) ^c^	86.5% (*p* < 0.001)	4	− 7.80 (− 10.64, − 4.96) ^d^	75.1% (*p* = 0.007)
Non-Asia	3	− 0.86 (− 1.33, − 0.40) ^b^	0.0% ((*p* = 0.502)	2	− 1.45 (− 2.25, − 0.65)	0.0% (*p* = 0.391)	2	− 3.08 (− 5.32, − 0.85)	0.0% (*p* = 0.411)

*WMD* weighted mean difference, *CI* confidence interval

^a^ The result of the subgroup was inconsistent with the overall analysis of WOMAC pain score for not achieving statistical significance

^b^ The effect size of the specific subgroups decreased to be lower than the MCID ( 1,18) of VAS for pain

^c^ The effect size of the specific subgroups increased to exceed the MCID (2.12) of WOMAC pain score

^d^ The effect size of the specific subgroups increased to exceed the MCID (6.62) of WOMAC function score

### Publication bias

The Egger’s linear regression test for VAS for pain, WOMAC total score, WOMAC pain score, WOMAC function score, WOMAC stiffness score and AEs did not detect significant publication bias (*P* = 0.318, 0.96, 0.78, 0.515,0.63 and 0.179 respectively), however, asymmetry of funnel plots was observed by visual inspection, which indicating the existence of potential publication bias (Supplementary Fig. 3).

## Discussion

The principal finding of our study was that CURs were associated with better effectiveness than placebo and not inferior to NSAIDs in terms of pain reduction and functional promotion for knee OA. The pooled analyses found that CURs were more effective than placebo in the improvement of VAS for pain, WOMAC total score, WOMAC pain score, WOMAC function score and WOMAC stiffness score, while there was no significant difference found between CURs and NSAIDs. We used the MCID as a threshold in this meta-analysis to assess the clinical significance of the difference between CURs and the control groups, instead of rely solely on the statistical significance. The MCID can be calculated by anchor-based and distribution-based methods, we applied the anchor-based method to set the threshold at 20% based on previous research [27, 33–36]. The significance test of clinical benefits found that only VAS for pain and WOMAC total score achieved clinical significance by exceeding their MCID, while WOMAC pain score, WOMAC function score and WOMAC stiffness score did not. We also found that CURs did not induce an increase of AEs compared with placebo and NSAIDs. The total incidences of AEs in CURs and control groups were 25.06% and 35.57%. Diarrhea and/or constipation and stomach pain (5.8% and 8.17%) were the most frequent mild AEs in CURs and control groups respectively (Table 4).

Pain and dysfunction were the leading causes for medical care use and clinical decision making for knee OA [73]. Novel disease-modifying treatments targeting the pathological process of OA are in development to solve the treatment dilemma of symptom-relieving drugs (pain-killers or NSAIDs) [74]. Among which, CURs have attracted much attention of medical researchers and clinicians [8–10]. CURs have been shown to possess therapeutic effects on knee OA as a result of their anti-inflammatory and anti-oxidant properties [15]. The regulation of inflammation- and catabolism-related pathways is the main mechanism underlying the anti-inflammatory and chondroprotective properties of CURs [75]. CURs exhibit anti-apoptotic and antioxidant effect on chondrocytes and induce mesenchymal stem cells chondrogenic proliferation. Thus far, many pre-clinical and clinical studies [76] have identified CURs as being effective for treating knee OA. Despite the highly pleiotropy in knee OA, the application of CURs is controversial due to poor oral bioavailability. Numerous studies have focused on methods to optimize the pharmacokinetics of CURs [77]. Among the included studies, ten used bioavailable CURs, such as nanocurcumin [61, 70, 71], liposome CURs complexes [58, 67] and so on, while the other five used pure extracts from CL. Theoretically, bio-optimized CURs should be superior to pure extracts given their higher absorptivity and lower metabolism [77]. According to our study, we found that the result of the bio-optimized extracts subgroup increased to exceed the MCID of WOMAC pain score, but the result of the pure extracts subgroup was neither statistically nor clinically significant. Besides, the effect sizes of both VAS for pain and WOMAC function score in the bio-optimized extracts subgroup exceeded their MCID, while those in the pure extracts group did not. These findings indicated that bio-optimized CURs may have better clinical applicability for knee OA than pure CURs. However, a recent meta-analysis of Wang et al. [24] found no significant difference between the enhanced and normal CL extracts in pain and physical function related outcomes. Two trials [44, 59] applying normal CURs as adjuvants to NSAIDs were included in their placebo-controlled group for quantitative synthesis, which may cause the divergence in the priority of the enhanced CURs given that the added effects of NSAIDS were neglected. Each study in this meta-analysis applied different metrics and tactics to remodel the bioavailability of CURs, direct comparisons between different CURs products are essential to verify our findings and seek a cost-effective agent. Predictably, bio-optimization techniques with more than one approach to conquer the hindrances (e. g., poor water solubility, rapid metabolism, and instability) to oral bioavailability would achieve significant improvement in the effectiveness of CURs.

The appropriate dosage of CURs for treating knee OA remains uncertain. Previous meta-analyses by Daily et al. [10] and Onakpoya et al. [9] demonstrated the typical dose of 1,000 mg/day as effective in the improvement of inflammation-related symptoms. According to our study, there was no statistically significant difference in the main outcomes between CURs and placebo in all subgroups of different doses (daily dose < or ≥ 1,000 mg and total dose < or ≥ 50 g). Theoretically, the optimal dose of a drug is closely associated with its safety and bioavailability. Various studies focused on diverse diseases have proved that CURs are effective without major safety concerns even at high doses such as 6 g/day [78], which indicated that the main limitation of optimal dosage is the bioavailability of CURs. Thus theoretically, the requirement for CURs of lower dosage and better compliance without affecting curative effects for knee OA may be met by the optimization of bioavailability. Actually, we found that the effect sizes of VAS for pain, WOMAC pain score and WOMAC function score in low-dose (daily dose < 1,000 mg and total dose < 50 g) subgroup exceeded their MCID, while those in high-dose (daily dose ≥ 1,000 mg and total dose ≥ 50 g) subgroup did not achieve clinical significance. The observed difference in clinical values between low- and high-dose subgroup may be caused by the limited number of studies in each subgroup or the administration of bio-optimized CURs for all studies in low-dose group.

A recently published review by Zeng et al. [26] suggested that CURs could not exhibit significant therapeutic effects until the duration of administration lasted for more than 12 weeks. Given the simultaneous inclusion of active-controlled [44, 56, 57, 59, 79] and placebo-controlled trials in their subgroup analyses, the effect sizes at different time points may be weakened by effects of NSAIDs-controlled arms. Thus, we removed the data of active-controlled trials in subgroup analyses, and found that CURs showed favorable improvement in VAS for pain, WOMAC pain score, and WOMAC function score compared to placebo at each time points (follow-up duration < or ≥ 12 weeks). Besides, the MCID was exceeded by the effect sizes of all main outcomes in the time < 12 weeks subgroup, but the effect sizes of the WOMAC pain score and WOMAC function score in the time ≥ 12 weeks subgroup did not achieve clinical significance. The reason for the difference in clinical values between short- and long-term subgroup may be that all three studies [62, 67, 71] in the short-term subgroup applied bio-optimized CURs, and two [65, 69] of the three [65, 66, 69] studies in long-term subgroup used normal CURs. Overall, in terms of alleviating pain and other symptoms, bio-optimized CURs may be sufficiently potent to lower dosage and shorten medication cycle. Besides, larger effect sizes with clinical significance of both pain reduction and functional promotion were observed in trials performed in Asia compared with those in other countries, which was in accordance with the result of a recently published review by Wang et al. [24].

The quality of our findings was evaluated using the GRADE system [37]. All pain and function related outcomes were downgraded to have a moderate to very low quality of evidence duo to inconsistency, risk of bias, and publication bias, while AEs were defined as high-quality evidence (Supplementary Table 3). The US Food and Drug Administration (FDA) defined CURs as nutraceuticals under “Generally Recognized as Safe” (GRAS) [80], and good safety and tolerability properties have been revealed by multitudinous studies at cellular level, in animals and even in human subjects [78], but it is still worth noting that nutraceuticals like CURs generally lack a systemic safety assessment before being used for medicinal purposes [81],therefore the potential dose- and time-dependent side effects of CURs on human body should be weighted carefully when facing the current benefits and potential values for broader clinical use of CURs.

### Strengths and limitations

In this study, we included the latest fifteen clinical trials focused on this topic. Meanwhile, trials with CURs-free or combined interventions were excluded to realize a more objective display of the therapeutic effect of CURs alone for knee OA. Besides, the clinical significance of CURs in alleviating pain and dysfunction for knee OA was also evaluated by the MCID of patient-reported outcomes. However, the limitations of our study should be considered when interpreting our findings. Firstly, the quality of the original studies was low, and substantial heterogeneity was detected among the included studies, and the exact sources of heterogeneity were hard to be found for which may stem from the multitudinous variations in dosages, follow-up durations, regions, preparation schemes of CURs, and baseline values. Secondly, obvious heterogeneity was still present after subgroup analyses, which indicated that the substantial heterogeneity was not entirely stem from the subgrouping variables. The quality of the included studies was uneven, the degree of bias was large and the numbers of studies in each subgroup was rather small, which could result in large differences in the results of statistical analysis. Thirdly, the durations of follow-ups in main outcomes were limited to within 6 months, as a result, the long-term clinical effectiveness of CURs remains equivocal. Although Egger's test did not indicate significant publication bias, the limited data volume of included studies made the linearity assessment quite uncertain and at risk of being overly influenced by single studies, and the asymmetry of funnel plots observed by visual inspection also indicated the existence of potential publication bias. Because the symmetry of funnel plots could be influence by various factors, such as publication bias, and/or small-study effects, it was difficult to figure out the cause of asymmetry, especially when the sample size of each comparison was less than ten [82]. For the reasons above, further studies are needed to warrant our findings and perform more comprehensive analyses.

## Conclusion

CURs alone can be expected to achieve considerable analgesic and functional promotion effects for patients with symptomatic knee OA in short-term, without inducing an increase of adverse events. However, considering the low quality and substantial heterogeneity of present studies, a cautious and conservative recommendation for broader clinical use of CURs should still be made. Further high-quality studies are necessary to investigate the impact of different dosages, optimization techniques and administration approaches on long-term safety and efficacy of CURs, so as to strengthen clinical decision making for patients with symptomatic knee OA.

## Supplementary Information

**Additional file 1.**

**Additional file 2.**

**Additional file 3.**

**Additional file 4.**

## Data Availability

All data generated or analyzed during this study are included in this article [and its supplementary information files].
