# Supplementary material for: Efficacy and safety of curcuminoids alone in alleviating pain and dysfunction for knee osteoarthritis: a systematic review and meta-analysis of randomized controlled trials
Source: BMC Complement Med Ther. 2022 Oct 19;22:276. doi: 10.1186/s12906-022-03740-9 (PMC9580113; doi:10.1186/s12906-022-03740-9)
Supplement: Supplementary file 2 — Additional file 2. [file 12906_2022_3740_MOESM2_ESM.docx]

**Supplementary Table 2** Retrieval strategy sample of PubMed and Embase

**Pubmed**

| #1 | osteoarthritis, knee [MeSH terms] |
| --- | --- |
| #2 | Osteoarthritis [MeSH terms] |
| #3 | knee osteoarthritis [text word] |
| #4 | KOA [text word] |
| #5 | OA [text word] |
| #6 | Or/#1-5 |
| #7 | Curcumin [MeSH terms] |
| #8 | Curcuma [MeSH terms] |
| #9 | curcuminoids [text word] |
| #10 | demethoxycurcumin [text word] |
| #11 | bisdemethoxycurcumin [text word] |
| #12 | tumeric [text word] |
| #13 | diferuloylmethane [text word] |
| #14 | Or/#7-13 |
| #15 | Randomized Controlled Trial [MeSH terms] |
| #16 | controlled clinical trial [text word] |
| #17 | randomized [text word] |
| #18 | Or/#15-17 |
| #19 | #6 and #14 and #18 |
| ("osteoarthritis, knee"[MeSH Terms] OR "osteoarthritis"[MeSH Terms] OR "knee osteoarthritis"[Text Word] OR "KOA"[Text Word] OR "OA"[Text Word]) AND ("curcumin"[MeSH Terms] OR "curcuma"[MeSH Terms] OR "curcuminoids"[Text Word] OR "demethoxycurcumin"[Text Word] OR "bisdemethoxycurcumin"[Text Word] OR "tumeric"[Text Word] OR "diferuloylmethane"[Text Word]) AND ("randomized controlled trials"[MeSH Terms] OR "controlled clinical trial"[Text Word] OR "randomized"[Text Word]) | |

**Embase**

| #1 | 'knee osteoarthritis'/exp OR 'knee osteoarthritis |
| --- | --- |
| #2 | 'osteoarthritis' |
| #3 | koa |
| #4 | oa |
| #5 | Or/#1-4 |
| #6 | 'curcumin' |
| #7 | 'curcuma' |
| #8 | 'curcuminoid' |
| #9 | 'demethoxycurcumin' |
| #10 | bisdemethoxycurcumin |
| #11 | tumeric |
| #12 | diferuloylmethane |
| #13 | Or/#6-12 |
| #14 | 'randomized controlled trial' |
| #15 | 'controlled clinical trial' |
| #16 | randomized |
| #17 | Or/#14-16 |
| #18 | #5 and #13 and #17 |
