# Supplementary material for: Efficacy and safety of curcuminoids alone in alleviating pain and dysfunction for knee osteoarthritis: a systematic review and meta-analysis of randomized controlled trials
Source: BMC Complement Med Ther. 2022 Oct 19;22:276. doi: 10.1186/s12906-022-03740-9 (PMC9580113; doi:10.1186/s12906-022-03740-9)
Supplement: Supplementary file 3 — Additional file 3. [file 12906_2022_3740_MOESM3_ESM.pdf]

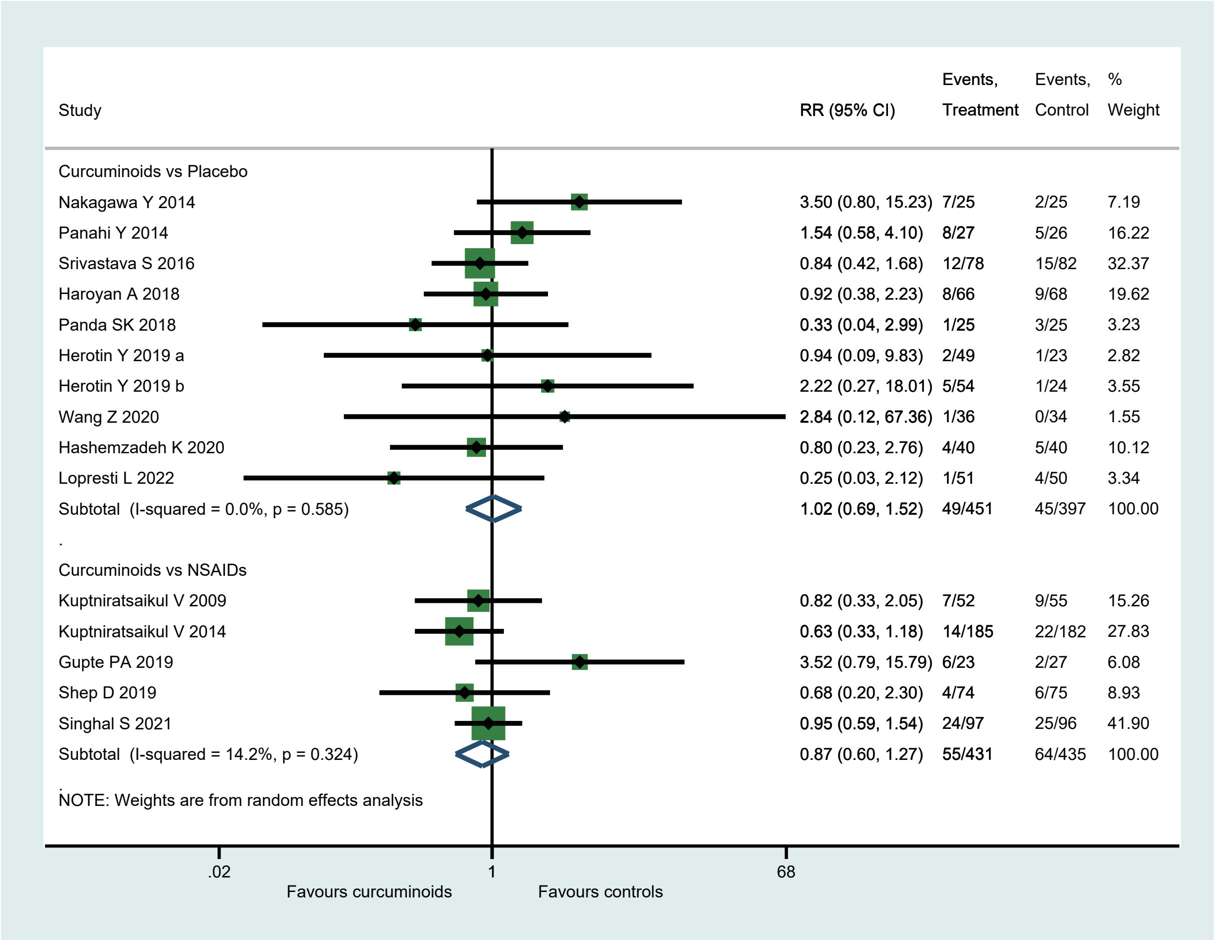

Supplementary Figure 1. Forest plot portraying the risk ratio with 95% confidence interval of withdraw rate.

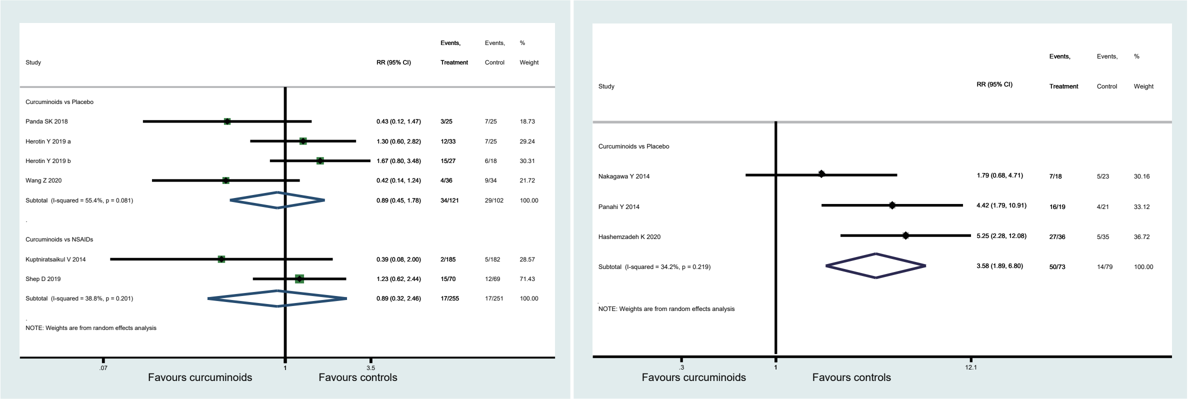

(A) (B)

Supplementary Figure 2. Forest plot portraying the risk ratio with 95% confidence interval of (A) use rate and (B) discontinuation rate of rescue medications.

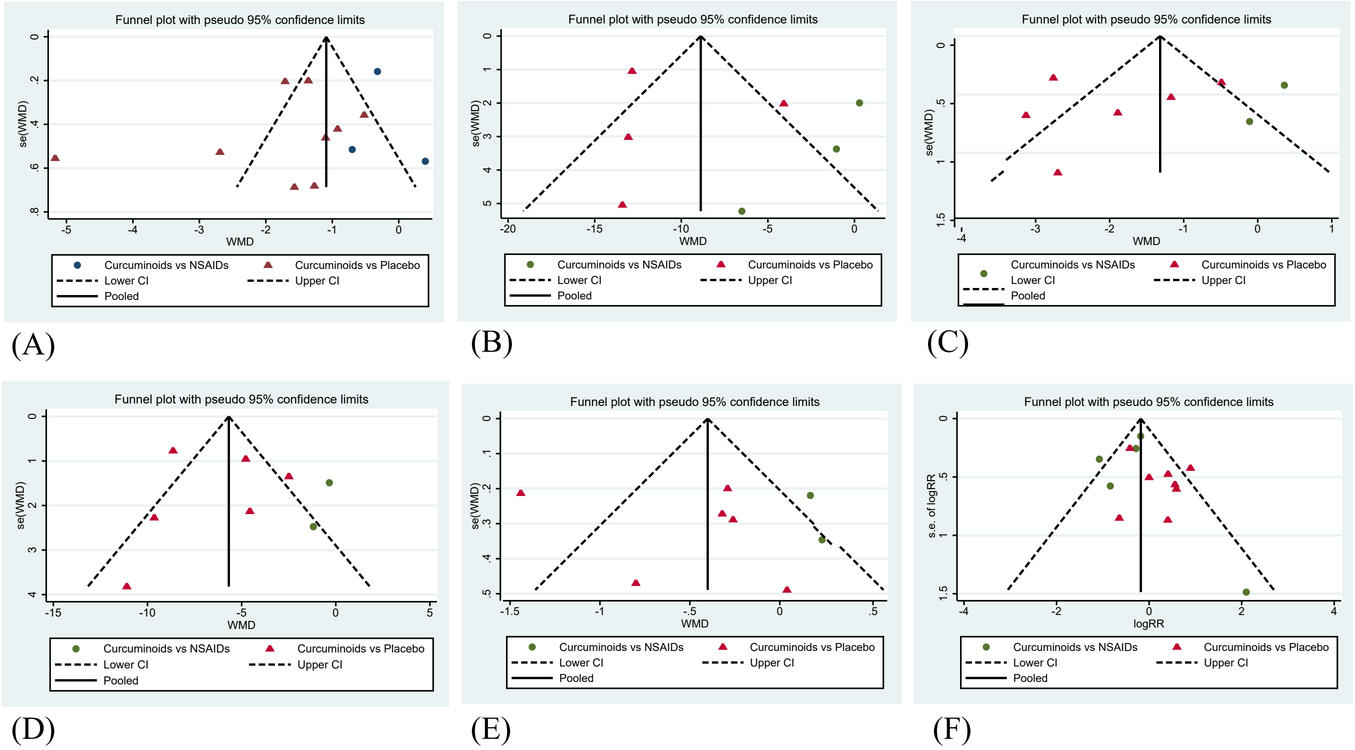

Supplementary Figure 3. Funnel plots of publication bias: (A) VAS for pian; (B) WOMAC total score; (C) WOMAC pain score; (D) WOMAC function score; (E) WOMAC stiffness score; (F) adverse events.
