# Supplementary material for: Efficacy and safety of curcuminoids alone in alleviating pain and dysfunction for knee osteoarthritis: a systematic review and meta-analysis of randomized controlled trials
Source: BMC Complement Med Ther. 2022 Oct 19;22:276. doi: 10.1186/s12906-022-03740-9 (PMC9580113; doi:10.1186/s12906-022-03740-9)
Supplement: Supplementary file 4 — Additional file 4. [file 12906_2022_3740_MOESM4_ESM.docx]

**Supplementary Table 3** GRADE quality assessment of evidence

| **Outcomes** | **No of Participants (studies) Follow up** | **Quality of the evidence** (GRADE) | **Relative effect (95% CI)** | **Anticipated absolute effects** | |
| --- | --- | --- | --- | --- | --- |
|  |  |  |  | **Risk with Control** | **Risk difference with CUR versus controls** (95% CI) |
| **Curcuminoids versus placebo** | | | | | |
| **VAS for pain** | 598 (9 studies) | ⊕⊕OO **LOW**^1^ due to inconsistency |  |  | The mean VAS for pain in the intervention groups was **1.77 lower** (2.44 to 1.09 lower) |
| **WOMAC total score** | 278 (4 studies) | ⊕⊕OO **LOW**^2^ due to inconsistency |  |  | The mean WOMAC total score in the intervention groups was **10.47 lower** (15.65 to 5.3 lower) |
| **WOMAC pain score** | 481 (6 studies) | ⊕⊕OO **LOW**^3^ due to inconsistency |  |  | The mean WOMAC pain score in the intervention groups was **1.94 lower** (2.91 to 0.97 lower) |
| **WOMAC function score** | 481 (6 studies) | ⊕⊕OO **LOW**^4^ due to inconsistency |  |  | The mean WOMAC function score in the intervention groups was **6.36 lower** (8.94 to 3.77 lower) |
| **WOMAC stiffness score** | 481 (6 studies) | ⊕⊕OO **LOW**^5^ due to inconsistency |  |  | The mean WOMAC stiffness score in the intervention groups was **0.54 lower** (1.03 lower to 0.05 higher) |
| **Adverse events** | 769 (9 studies) | ⊕⊕⊕⊕ **HIGH** | **RR 1.07**  (0.7 to 1.65) | **Study population** | |
|  |  |  |  | **156 per 1000** | **11 more per 1000** (from 47 fewer to 102 more) |
|  |  |  |  | **Moderate** | |
|  |  |  |  | **130 per 1000** | **9 more per 1000** (from 39 fewer to 84 more) |
| **Curcuminoids versus NSAIDs** | | | | | |
| **VAS for pain** | 272 (3 studies) | ⊕OOO **VERY LOW**^6^ due to risk of bias, publication bias |  |  | The mean VAS for pain in the intervention groups was **0.3 lower** (0.63 lower to 0.04 higher) |
| **WOMAC total score** | 517 (3 studies) | ⊕⊕⊕O **MODERATE**^7^ due to publication bias |  |  | The mean WOMAC total score in the intervention groups was **0.68 lower** (3.88 lower to 2.52 higher) |
| **WOMAC pain score** | 475 (2 studies) | ⊕⊕⊕O **MODERATE**^8^ due to publication bias |  |  | The mean WOMAC pain score in the intervention groups was **0.24 lower** (0.47 lower to 0.96 higher) |
| **WOMAC function score** | 475 (2 studies) | ⊕⊕⊕O **MODERATE**^9^ due to publication bias |  |  | The mean WOMAC function score in the intervention groups was **0.57 lower** (3.07 lower to 1.94 higher) |
| **WOMAC stiffness score** | 475 (2 studies) | ⊕⊕⊕O **MODERATE**^10^ due to publication bias |  |  | The mean WOMAC stiffness score in the intervention groups was **0.19 lower** (0.17 lower to 0.56 higher) |
| **Adverse events** | 800 (5 studies) | ⊕⊕⊕O **MODERATE**^11^ due to inconsistency | **RR 0.65**  (0.41 to 1.03) | **Study population** |  |
|  |  |  |  | **307 per 1000** | **107 fewer per 1000** (from 181 fewer to 9 more) |
|  |  |  |  | **Moderate** |  |
|  |  |  |  | **357 per 1000** | **125 fewer per 1000** (from 211 fewer to 11 more) |

*CI* Confidence interval; *RR* Risk ratio.

High quality: Further research is very unlikely to change our confidence in the estimate of effect.
Moderate quality: Further research is likely to have an important impact on our confidence in the estimate of effect and may change the estimate.
Low quality: Further research is very likely to have an important impact on our confidence in the estimate of effect and is likely to change the estimate.
Very low quality: We are very uncertain about the estimate.

^1^ Inter-study heterogeneity of studies was 86.8%, which was higher than 75% and equal to ‘very serious’ quality downgrade (minus 2).
^2^ Inter-study heterogeneity of studies was 80.6%, which was higher than 75% and equal to ‘very serious’ quality downgrade (minus 2).
^3^ Inter-study heterogeneity of studies was 79.2%, which was higher than 75% and equal to ‘very serious’ quality downgrade (minus 2).
^4^ Inter-study heterogeneity of studies was 79.2%, which was higher than 75% and equal to ‘very serious’ quality downgrade (minus 2).
^5^ Inter-study heterogeneity of studies was 77.6%, which was higher than 75% and equal to ‘very serious’ quality downgrade (minus 2).

^6^ Kuptniratsaikul V 2014 (Ref. 57) and Shep D 2019 (Ref. 67) did not apply adequate blinding methods (minus 2); and the number of studies is limited, publication bias is likely to exist (minus 1).

^7, 8, 9, 10^ The number of studies is limited, publication bias is likely to exist (minus 1).

^11^ Inter-study heterogeneity of studies was 55.8%, which was higher than 50% and equal to ‘serious’ quality downgrade (minus 1).
